# Fisetin Ameliorates Diabetic Nephropathy-Induced Podocyte Injury by Modulating Nrf2/HO-1/GPX4 Signaling Pathway

**DOI:** 10.1155/2023/9331546

**Published:** 2023-01-19

**Authors:** Xiaojing Qian, Shan Lin, Ji Li, ChengLin Jia, Yun Luo, Rui Fan, Cheng Hu, Ying Chen

**Affiliations:** ^1^Shanghai TCM-Integrated Hospital Affiliated to Shanghai University of Traditional Chinese Medicine, Shanghai 200082, China; ^2^Shanghai University of Traditional Chinese Medicine, Shanghai 201203, China; ^3^Jiading Branch of Shanghai General Hospital, Shanghai Jiao Tong University School of Medicine, Shanghai 201803, China

## Abstract

Diabetic nephropathy (DN) is one of the most severe microvascular complications of diabetes and has become the leading cause of end-stage renal disease formation. The pathogenesis of diabetic nephropathy is very complex and is still not fully understood. Fisetin is a flavonoid polyphenolic compound that is widely found in different fruits, vegetables, and medicinal plants. Many studies have indicated that it has a variety of pharmacological activities. In this study, we investigated the mechanism of action of fisetin in the protection of DN-induced podocyte injury both in vivo and in vitro. Results showed that fisetin could reduce high glucose (HG)-induced podocyte injury and streptozotocin (STZ)-induced diabetic nephropathy in mice. According to the histopathological staining results, fisetin ameliorated DN-induced glomerular injury in a dose-dependent manner. Western blot and immunofluorescence results showed that fisetin effectively promoted the expression of podocyte functional integrity marker proteins and inhibited the expression of podocyte injury marker proteins. In addition, according to the Western blot and RT-qPCR results, fisetin activates the nuclear translocation of Nrf2 to exert antioxidative stress ability and affects the expression of downstream antioxidant enzymes HO-1, GPX4, and other ferroptosis-related markers, thereby protecting against HG-induced podocyte ferroptosis and oxidative stress injury in DN mice. In summary, this study demonstrated that fisetin could enhance the antioxidative stress capacity of DN mice by promoting the activation of the Nrf2/HO-1/GPX4 signaling pathway in renal tissues, and attenuated HG-induced podocytes injury and STZ-induced DN in mice.

## 1. Introduction

The incidence of DN in China has increased dramatically over the past decade, and it has become the leading cause of end-stage renal disease worldwide owing to severe metabolic disorders [1]. Until now, DN has not been fully understood because of its complex pathogenesis. The typical pathological changes of DN are mainly manifested in glomerular and tubular basement membrane thickening, thylakoid stromal hyperplasia, and tubulointerstitial fibrosis, resulting in increased urinary protein excretion and gradually leading to renal failure [2].

Podocytes are important intrinsic cells in renal tissue, and previous studies have found that structural integrity and a constant number of podocytes maintain the normal filtration capacity of the kidney [3]. In contrast, a decrease in the number of podocytes can lead to symptoms such as glomerulosclerosis and renal failure. Therefore, interventional therapy for podocyte death and inhibition of podocyte apoptosis are essential for the treatment of DN.

Due to a long-term high glucose environment, DN patients are prone to produce excessive ROS clusters in the body, which will lead to significant injuries to the kidney [4]. According to relevant studies, the Nrf2/HO-1 signaling pathway is strongly associated with oxidative stress-related diseases [5]. Landis et al. [6] found that Isoeucommin A alleviated inflammation and oxidative stress both in a high-glucose-stimulated human renal thylakoid model in vitro and in vivo DN model, thereby reducing oxidative stress by activating the Nrf2/HO-1 signaling pathway to attenuate renal injury. Li et al. [7] suggested that the Nrf2/HO-1 pathway may confer cytoprotective function and ameliorate diabetic nephropathy by restoring the M1/M2 macrophage conversion mechanism.

In 2003, Stockwell B. R.'s research team discovered a programmed cell death process that is different from apoptosis and autophagy during the experimental screening of anticancer drugs [8]. In 2012, they named this new form of cell death due to Fe^2+^-dependent lipid peroxide accumulation as ferroptosis [9]. Ferroptosis manifests biochemically as an increase in iron ion-dependent lipid peroxides and ROS [10]. Current studies have found that ferroptosis is associated with numerous physiological and pathological processes, such as tumor proliferation, atherosclerosis, neurodegenerative diseases [11], diabetes, acute kidney injury (AKI), ischemia-reperfusion injury [12], and cancers [13]. Wang et al. [14] showed for the first time that ferroptosis is involved in the process of renal tubular cell death in DN and is also involved in the progression of DN both in vivo and in vitro, which may be a future direction for DN treatment. Another study demonstrated that ferroptosis plays an important role in the development of DN, and upregulating Nrf2 by treating with fenofibrate inhibited diabetes-associated ferroptosis, shedding light on the mechanism of DN development from a new perspective and providing a new approach to delay the occurrence and development of this disease [4]. Therefore, further exploration of ferroptosis pathways and interventional drugs has become a current research hotspot in DN.

Fisetin (3,7,3′,4′-tetrahydroxyflavone, FST) is a flavonoid polyphenolic compound widely found in fruits, vegetables, and medicinal plants [15]. Ge et al. [16] showed that fisetin can prevent DN by inhibiting insulin resistance and the RIP3-regulated inflammatory response. A previous study of our group confirmed that FST attenuates high glucose-induced podocyte injury and ameliorates STZ-induced DN in mice by restoring the autophagy-mediated CDKN1B/p70S6K pathway and inhibiting NLRP3 inflammatory vesicles [17]. Ehrens' group [18] found that FST rapidly promotes the expression of Nrf2 and activates transcription factor 4, thereby scavenging free radical activity and improving antioxidant capacity. They also suggested FST has various pharmacological effects such as cardioprotection, neuroprotection, antiaging, improvement of nonalcoholic fatty liver, and treatment of diabetes mellitus. Moreover, related studies have found that FST can inhibit ferroptosis by activating the SIRT1/Nrf2 signaling pathway and attenuate Adriamycin-induced cardiomyopathy in vivo and in vitro [19]. However, no studies have confirmed the relationship between FST and the Nrf2/HO-1 pathway in DN.

In this study, in order to investigate the effect of FST on the remission of DN, we used eNOS pure knockout (eNOS-/-) mice with intraperitoneal injection of streptozotocin to verify and compare the effects of different doses of fisetin on the expression of ferroptosis marker protein in DN mice, and its effects on the expression of antioxidant enzymes and cytokines in renal tissues. We also explored the mechanism by which fisetin interacts with the Nrf2/HO-1 signaling pathway.

## 2. Materials and Methods

### 2.1. Reagents and Chemicals

Fisetin was purchased from Shanghai Yuan ye Biotechnology Co. Ltd (Catalog No. B20953, purity ≥ 98%); Streptozotocin (STZ) was from Sigma-Aldrich (Catalog No. S0130, Sigma-Aldrich, St. Louis, MO, USA); HE staining kit, (lot no. G1003), PAS staining kit, (lot no. G1006), both purchased from Servicebio, China; CCK-8 cell viability kit, Bebe Bio (item no. BB4202); Urine Protein Test Kit (cat. no. C035-2), Creatinine Assay Kit (cat. no. C011-2), Glutathione Peroxidase (GSH) assay kit (cat. no. A005-1-2), Malondialdehyde (MDA) assay kit (cat. no. A003-1-2), and Superoxide Dismutase assay kit (cat. no. A001-3-2); Nanjing Jian Cheng Bioengineering Institute, Nanjing, China); HO-1 antibody, Proteintech, China, lot: 27282-1-AP, was purchased from Cell Signaling Technology (lot: 5174, #82206); Nrf2, GPX4, and ACSL4 were purchased from Abcam, USA (lot: ab137550, ab12500); Desmin was purchased from Proteintech (item no. 60226-1-lg); Podocin was purchased from Santa Cruz (item no. sc-518088); Rabbit anti-Lamin B antibody, lot no. bs-24328R; rabbit anti-*β*-actin Antibody, China BIOSS, Lot No: bs-0061R; BCA Kit, Biological Industries (Item No: P0010S); Fetal Bovine Serum, Biological Industries (Item No: 2022057); Horseradish Peroxidase-Labeled Goat Anti-Mouse lgG, Horseradish Peroxidase-Labeled Goat Anti-Rabbit lgG. Proteintech; Two Step Reverse Transcription Kit, Vazyme (Item #: R323-01); SYBR qPCR Kit, Vazyme (Item #: Q711-02); TRIzol Kit, In-vitrogen; RIPA Protein Lysate (Lot #: P0013B); Cell Nucleus Protein and Cell Plasma Protein Extraction Kit (lot no.: P0028), purchased from Beyoncé Biotechnology, China.

### 2.2. Instruments

Flow cytometer (Beckman Coulter, Inc.); enzyme labeler (Molecular Devices, Inc.); gel imaging system (Peiqing, Inc.); fluorescence inverted/anterior microscope (Leica, Inc.); cellSens Standard image acquisition software (Olympus, Japan); laser confocal microscope (Leica, Inc.); fluorescence quantification ABI7500 (Thermo Fisher Scientific, Inc.); cryogenic high-speed centrifuge (Sigma, USA); electrophoresis instrument and electrophoresis tank (Bio-Rad, USA); fluorescence quantitative ABI7500 (Thermo Fisher Scientific); cryogenic high-speed centrifuge (Sigma, USA); electrophoresis instrument and electrophoresis tank (Bio-Rad, USA); and nLC 1000-Orbitrap Fusion mass spectrometer (Thermo Fisher Scientific).

### 2.3. Experimental Animals and Experimental Design

eNOS homozygous knockout (eNOS-/-) mice (male, 18–22) (background C57BL/6J) were housed under specific pathogen-free conditions. Our study was carried out in strict accordance with the Guide for the Care and Use of Laboratory Animals (Eighth Edition, 2011, published by The National Academies Press, 2101 Constitution Ave. NW, Washington, DC 20055, USA). The protocol was reviewed and approved by the Animal Care Committee of Shanghai TCM-Integrated Hospital (Permit Number: PZSHUTCM201204008).

30 mice were randomly divided into 5 groups (*n* = 6). Normal control group was injected intraperitoneally with citrate buffer solution. In the HG model group, 8-week-old male eNOS homozygous knockout (eNOS-/-) mice were given intraperitoneal injection (Ip) of 1% STZ solution (STZ dissolved in 0.1 mmol/l citric acid buffer, pH 4.5) to induce diabetes, the mice were fasted for 4–6 h daily and injected IP (50 mg/kg, daily) for consecutive 5 days [14, 17, 20], when the fasting blood glucose levels from tail vein of STZ-induce diabetic mice were over 16.7 mM at 5 days after STZ injection, these mice were considered as diabetes. In different concentrations of FST group, knockout mice were given different doses of FST (5, 10, 20 mg/kg body weight) [17] by gavage once daily for 8 weeks after 10 weeks of diabetes induction. After 18 weeks of diabetes onset, the urine of the rats was collected over the 24 h prior to sacrifice using metabolic cages. Mice were anaesthetized with isoflurane and blood samples were taken from the inferior vena cava and sacrificed. The serum was collected and centrifuged at 860 g for 15 min at 4°C. The kidneys were surgically harvested, and part of them were used for histopathological examination, while the remaining part of kidney cortex was chopped and immediately placed in liquid nitrogen and transferred to −80°C for protein detection. The surgery was performed under sodium pentobarbital anesthesia, and all efforts were made to minimize pain.

### 2.4. Histopathological Examination

The kidney tissues were taken, and the right kidney was longitudinally cut and fixed in 4% paraformaldehyde for 24 hours. The sections were embedded in paraffin and sectioned for H&E (hematoxylin-eosin) staining and PAS (periodic acid-schiff) staining, respectively. After routine treatment, the histopathological changes in the kidney tissues of each group of mice were observed under the microscope.

### 2.5. Laser Confocal Microscopy Assay

Differentiated mature podocytes were cultured in laser confocal dishes; the cells were fixed at room temperature with 4% paraformaldehyde, washed 3 times with PBS, blocked with 3% BSA for 30 min, and incubated with desmin primary antibody (1 : 500) overnight at 4°C. The next day, cells were incubated with secondary antibody (1 : 200) for 2 h at room temperature, followed by DAPI staining for 10 min and PBS washing and then ready for observation under laser confocal microscopy.

### 2.6. Determination of FBG, 24-h Urine Protein, and Serum Creatinine (SCr) Levels

The fasting blood glucose was obtained from the tail vein by using glucometers (Accu-Chek Active Blood Glucose Meter System) every week. At 10 weeks postinduction, the mice were placed in the metabolic cages for 24-h urine collection and consequent albuminuria measurement prior to being anaesthetized with isoflurane, and blood samples were taken from the inferior vena cava and sacrificed. Then, the blood was placed in serum tubes and centrifuged at 860 g for 15 min at 4°C. The serum was collected, and the 24-h urine protein and SCr were measured by using the Urine Protein Test Kit (cat. no. C035-2) and Creatinine Assay Kit (cat. no. C011-2; Jian Cheng Bioengineering Institute, Nanjing, Jiangsu, China) according to the manufacturer's protocols.

### 2.7. Determination of GSH, MDA, and SOD Levels

Renal tissues were removed and homogenized with cold PBS buffer to obtain 10% homogenate (w/v). The supernatant fluid was collected following centrifugation at 825 g for 20 min at 4°C. The renal GSH, MDA, and SOD levels were measured according to the protocols of the Glutathione Peroxidase assay kit (Colorimetric method) (cat. no. A005-1-2), Malondialdehyde (MDA) assay kit (cat. no. A003-1-2), and Superoxide Dismutase Assay kit (cat. no. A001-3-2); Nanjing Jian Cheng Bioengineering Institute, Nanjing, China).

### 2.8. RNA Extraction and RT-qPCR

The cells were collected according to the TRIzol extraction instructions, and the total RNA was stored at −80°C after determination of its concentration and purity. Follow the procedure of Vazyme kit R323-01 : 2 min at 42°C, 15 min at 37°C, 5 s at 85°C, and cDNA synthesis at 4°C; the product is used immediately for experiment or stored at −20°C. The reaction procedure was as follows: predenaturation, 95°C for 30 s, 95°C for 10 s, and 60°C for 30 s, 40 cycles; solubility curve was 95°C for 15 s, 60°C for 60 s, and 95°C for 15 s.

The ACSL4 primer sequence was F-CCCTGAAG-GATTTGAGATTCACA and R-CCTTAGGTCGGCCAGTAGAAC; the upstream primer of GPX4 was 5′-TGTGCATCCCGCGATGATT-3′, and the downstream primer was 5′-CCCTGTA CTTATC CAGGCAGA-3′. Three replicate wells were set up for each group, and the data were counted using the 2^−ΔΔCT^ method.

### 2.9. Proteomics Studies

The commercial FFPE-FASPTM protein digestion kit was used for protein extraction and digestion of kidney tissue proteins (Execion Inc., San Diego, CA). Sequencing grade trypsin (Promega, Madison, MI) was added to the filter for digestion. The devices were incubated overnight at 37°C, and the peptides were collected by centrifugation at 15,000 rpm for 10 min. Finally, peptide samples were desalted using a ZipTip C18 tip (Millipore, Billerica, MA) prior to LC-MS analysis.

For nano LC-MS/MS and data analysis, proteomics analysis was performed on an Accept PepMap RSLC C18 column (75 *μ*m × 25 cm, 2 *μ*m, Nano Viper, 100 A) using a nano flow HPLC Easy-nLC 1000 system (Thermo Fisher Science). The mobile phases were water-0.1% formic acid (A) and acetonitrile-0.1% formic acid (B). The gradients were set as follows: 0–2 min, 2–8% B; 2–112 min, 8–28% B; 112–114 min, 28–90% B; 114–120 min, 90% B.

Orbitrap Fusion Lumos mass spectrometers (Thermo Fisher Science) were used to perform proteomic analyses. The spray voltage in positive ion mode was 2100 V. The Orbitrap mass analyzer was used to acquire ions with m/z of 350–1600 at a resolution of 120,000. Precursor ions were fragmented with high collisional dissociation (HCD) and a normalized collision energy of 30%. The separation window was set at 0.7 m/z. The acquired spectral data were processed and analyzed with Proteome Discoverer 2.4SP1 software (Thermo Fisher Science).

### 2.10. Western Blot

To observe the effect of FST on the Nrf2/HO-1/GPX4 pathway in the kidneys of DN mice, Western blot was used to detect the expression of related proteins. The mouse kidney cortex was treated with RIPA lysate for total protein extraction and the nucleoplasmic protein extraction kit for nucleus and plasma protein extraction, respectively. The protein concentration was determined by a BCA kit. The main steps are as follows: first, the proteins were separated by electrophoresis using a 10% SDS-PAGE separation gel; second, the separated proteins were electrotransferred to PVDF membrane and blocked with 5% skimmed milk at room temperature for 2 hours; subsequently, they were incubated with primary antibody Nrf2 (1 : 1000 dilution), HO-1 (1 : 1000 dilution) and GPX4 (1 : 1000 dilution) overnight at 4°C; the next day, the rabbit secondary antibody (1 : 5000 dilution) was incubated at room temperature for 2 hours. Finally, the proteins were visualized on a chemiluminescence imager. Image J software was used to analyze the grayscale values of proteins, and Lamin B and *β*-actin were used as internal reference genes to calculate the relative expression levels of each group of proteins.

### 2.11. Statistical Analysis

SPSS 20.0 statistical software was used to analyze the experimental data, and all results were expressed as the mean ± standard deviation of each group. For multiple comparisons, one-way ANOVA followed by Tukey's test was used and *P* < 0.05 was considered statistically significant.

## 3. Results

### 3.1. FST Alleviates Kidney Damage in DN Mice

To establish a type I diabetic nephropathy mouse model, we induced diabetes in mice by intraperitoneal injection of (Ip) STZ [17].

**Figure 1:**
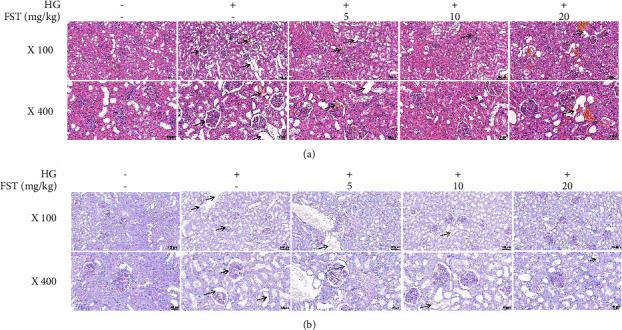
Histomorphological observation of kidney in each group of mice. (a) H&E staining of kidney sections (×100, 400); (b) PAS staining of kidney sections (×100, 400).

The results of HE staining are shown in Figure 1(a). The NC group mice had normal glomerular structure and neatly arranged renal tubules with a clear outline; no abnormality was found in the interstitium. Compared to the NC group, the glomeruli of the DN model group increased in size, the thylakoid region enlarged more obviously, and the interstitium was infiltrated by a small number of inflammatory cells. Compared with the model group, the glomeruli, tubules, and interstitium of the low-, medium-, and high-dose fisetin groups were more neatly arranged, the glomeruli thylakoid region enlarged, and the glomeruli increased in size. The improvement was more significant with increasing FST concentrations. Histological analysis of PAS-stained (Figure 1(b)) kidneys showed that FST treatment effectively attenuated glomerular hypertrophy and thylakoid matrix expansion in DN mice.

**Figure 2:**
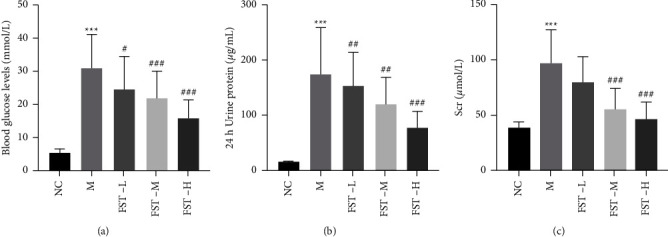
FST alleviates kidney damage in DN mice. (a) Blood glucose levels were measured in mice (*n* = 6). (b) 24 h urine protein levels in mice in the control, DN, and FST intervention at different dose (5, 10, 20 mg/kg) groups. (c) Serum creatinine levels in mice in the control, DN, and FST intervention at different dose (5, 10, 20 mg/kg) groups. Data are expressed as means ± SD. *n* = 6. ^*∗∗∗*^*p* < 0.001 compared to vehicle. ^#^*p* < 0.05 compared to DN. ^##^*p* < 0.01 compared to DN. ^###^*p* < 0.001 compared to DN. NC: normal control; M: DN model, STZ (50 mg/kg); FST-L: low dose of FST (5 mg/kg); FST-M: middle dose of FST (10 mg/kg); FST-H: high dose of FST (20 mg/kg).

As shown in Figures 2(a)–2(c), compared with the NC group, blood glucose levels, the 24 h urine protein level, and the Scr level in the model group were significantly increased, indicating that the DN model had been successfully established and a certain degree of kidney damage had occurred, but this effect gradually attenuated with the intervention of FST. Both results suggested that FST could effectively improve the abnormal kidney tissue morphology and alleviate tissue damage in DN mice.

### 3.2. Effect of FST on High Glucose-Induced Podocyte Marker Proteins

**Figure 3:**
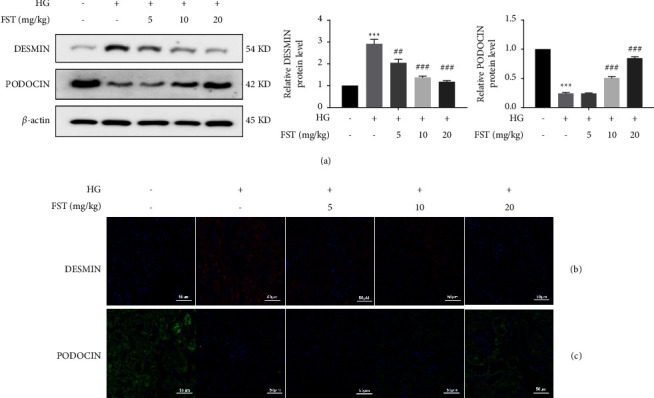
Effect of FST on the expression of podocyte marker proteins (*n* = 5). (a) Protein electrophoresis of each group of mice; DESMIN and PODOCIN protein level from each group of mice. Data are expressed as means ± SD. *n* = 6, ^*∗∗∗*^*p* < 0.001 compared to vehicle; ^##^*p* < 0.01 and ^###^*p* < 0.001 compared to DN. (b, c) Fluorescence staining of DESMIN and PODOCIN podocyte marker proteins.

Both desmin and podocin are marker proteins of podocytes [21]. Western blot results (Figure 3(a)) and laser confocal microscopy (Figures 3(b) and 3(c)) showed that, compared with the NC group, the expression of desmin in the podocytes of the DN group was significantly increased, suggesting a situation of podocyte damage. Compared with the DN model group, FST could significantly downregulate the expression of desmin with an increase in concentration, while the expression of podocin in the podocytes of the DN group was significantly decreased compared with the NC group. Compared with the DN model group, FST significantly upregulated the expression of podocin with increasing concentrations.

The above results demonstrated that FST effectively promoted the expression of podocin in HG-induced DN mouse podocytes and also inhibited the expression of desmin, indicating that FST could alleviate the podocytes damage and repair their function.

### 3.3. FST Inhibits Oxidative Stress in the Kidneys of DN Mice

**Figure 4:**
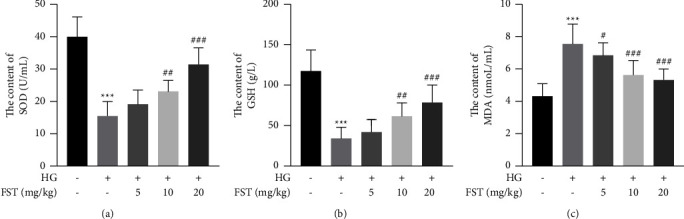
Determination of antioxidant factors in the serum of each group of mice (*n* = 8): (a) serum SOD content; (b) serum GSH content; (c) serum MDA content. Data are expressed as means ± SD (*n* = 8, ^*∗∗∗*^*p* < 0.001 compared to vehicle, ^#^*p* < 0.05, ^##^*p* < 0.01 and ^###^*p* < 0.001 compared to DN).

The results of this study showed that SOD and GSH levels were significantly lower (*P* < 0.001) and MDA levels were significantly higher (*P* < 0.001) in the DN model group compared with the NC group. However, FST could reverse these trends in a dose-dependent manner, decreasing MDA levels and increasing SOD and GSH activities in kidneys of DN mice (Figures 4(a)–4(c)).

### 3.4. Differentially Expressed Proteins (DEPs) Analysis

**Figure 5:**
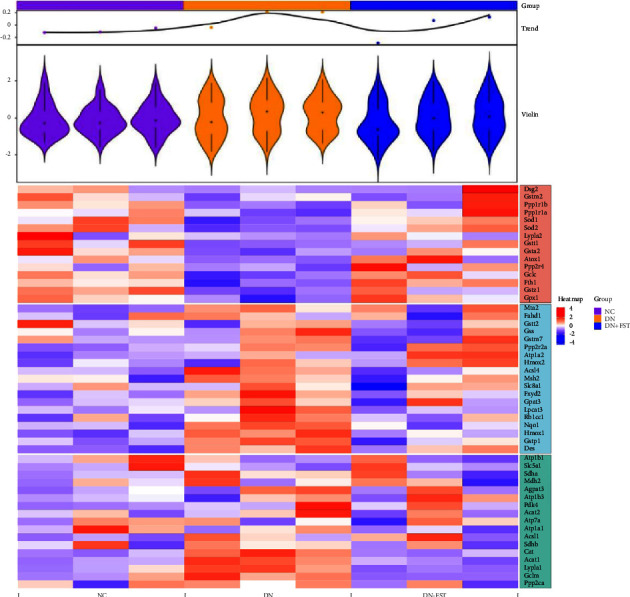
Heat map of differential proteins.

Quantitative proteomics was used to detect the differential protein expression in mouse kidney tissues. The heat map of the 51 differential genes obtained from the screening on the NC group, DN model group, and FST administration group is shown in Figure 5. The legend on the right side indicates the expression ploidy, with higher expression in red and lower expression in blue. Our results showed that the expression of Acsl4, Msh2, Gpat3, Nqo1, Hmox1, Gstp1, Cat, Acat1, Lypla1, and Gclm was significantly upregulated, while the expression of Ppp1r1a, Sod1, Sod2, Gsta2, Gclc, Fth1, and Gpx1 was significantly downregulated in the DN group compared to the NC group. However, Acsl4, Nqo1, Hmox1, Gstp1, Cat, Acat1, Lypla1, and Gclm showed a downregulation trend in the FST treatment group, while Sod1, Sod2, Gclc, Fth1, and Gpx1 expressions were increased, similar to the gene expression results in the NC group.

### 3.5. Effect of FST on Ferroptosis-Related Signaling Pathway Proteins in HG-Induced Podocytes

The Nrf2/HO-1 signaling pathway is closely related to ferroptosis and is a classic signaling pathway against oxidative stress [22]. The increase of reactive oxygen species in the body can cause damage to the organism. Nrf2 is a transcription factor that initiates endogenous antioxidant response elements [23], undergoes nuclear translocation in response to stimuli such as reactive oxygen species, and activates the expression of the antioxidant enzyme gene HO-1 to exert antioxidant stress capability, scavenging excess reactive oxygen species (ROS) and reducing podocyte death.

**Figure 6:**
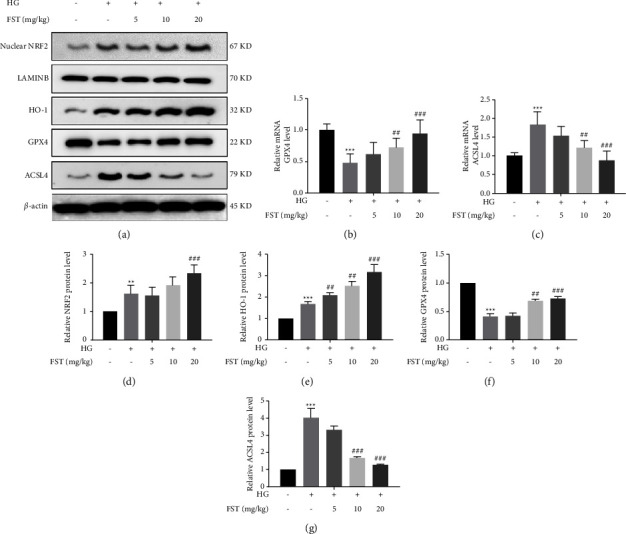
Effect of FST on ferroptosis-related signaling pathway proteins in high glucose-induced podocytes: (a) protein electrophoresis of each group of mice; (b) mRNA GPX4 levels of each group; (c) mRNA ACSL4 levels of each group; Nuclear NRF2 (d), HO-1 (e), GPX4 (f), ACSL4 (g) protein levels from mice of each group. Data are expressed as means ± SD (*n* = 6), ^*∗∗∗*^*p* < 0.001 compared to vehicle; ^##^*p* < 0.01 and ^###^*p* < 0.001 compared to DN).

Western blot results showed that, compared with the NC group, the expression of Nrf2 protein was increased in the podocyte nuclei of the mice in the DN group, which correspondingly triggered an increase in the expression of the downstream protein HO-1. This might be related to the protective response of the organism to oxidative stress damage, which is also consistent with the expression trend of HO-1 in the DN group (HG) of the differential metabolite heatmap. Compared with the DN model group, FST increased the nuclear translocation of Nrf2 and significantly upregulated the expression of the HO-1 protein with increasing concentration (Figure 6(a)). It was suggested that FST could activate the nuclear translocation of Nrf2 to exert antioxidative stress ability, affect the transcription of downstream antioxidant enzymes HO-1 and GPX4, and protect against ferroptosis injury in HG-induced podocytes (Figures 6(d)–6(g)).

The downregulation of GPX4 and elevation of ACSL4 suggest that ferroptosis may exist. RT-qPCR results showed that GPX4 levels were significantly downregulated and ACSL4 levels were significantly increased in the DN model group. Compared with the DN model group, with an increasing dose of FST, it significantly increased GPX4 levels and decreased ACSL4 levels, indicating that podocyte ferroptosis was alleviated (Figures 6(b) and 6(c)), and this result was also consistent with the western blot results and corresponding trend in the differential protein heatmap.

## 4. Discussion

DN is one of the serious complications of diabetes mellitus. Due to its serious metabolic disorders, it is the main cause of renal failure [24]. Therefore, early detection and intervention are vital for the prevention and treatment of DN, but there are no targeted therapeutic drugs available. Clinical treatment measures such as optimization of blood glucose, blood pressure, lipids, and weight control cannot completely prevent the progression of DN. Previous studies have shown that glomerular podocytes are the target cells for the action of high glucose [25], and the decrease in the number and structural damage of podocytes will lead to the destruction of the glomerular filtration barrier and then develop into glomerulosclerosis, renal failure, and other symptoms. Therefore, studying the mechanism of DN podocyte damage may provide new ideas for the intervention of this disease.

In the early stage, DN is insidious and may show pathological changes such as glomerular hypertrophy; in the middle stage, the disease progresses slowly and pathological changes such as thickening of glomerular and tubular basement membranes and marked hyperplasia in the thylakoid region may appear; in the later stage, glomerulosclerosis, interstitial fibrosis, and tubular atrophy may occur, which eventually lead to renal failure [26]. The results of our study showed that FST can reduce the degree of pathological changes such as glomerular volume increase, thylakoid zone hyperplasia, and basement membrane thickening, suggesting that it can obviously improve renal function and renal histopathology in DN and can delay the pathological process of renal fibrosis.

Podocin is an important protein for maintaining the functional integrity of podocytes [27], while the podocyte injury marker desmin protein is a marker of myogenic cells that is not significantly expressed in podocytes under normal conditions. When podocytes are subjected to persistent high glucose stimulation, a stress response occurs, resulting in a significant increase in reactive oxygen species in vivo and thereby causing damage to the organism. When the damage occurs, a large amount of desmin is expressed, further promoting the phenotypic transformation. In the present experiment, laser confocal microscopy showed that FST effectively inhibited desmin expression and also significantly increased podocin expression, demonstrating that FST can effectively alleviate oxidative stress damage in podocytes and repair podocyte function.

Nrf2, an important cytoprotective transcription factor in the Nrf2/HO-1 classical antioxidant stress signaling pathway [23], dissociates from Keap-1 in the cytoplasm when the organism undergoes oxidative stress. Then, it translocates to the nucleus, binds to antioxidant progenitors, and activates gene expression of downstream endogenous antioxidants such as HO-1, GPX4, etc., which act as a way to scavenge excess ROS [28]. In the present study, Western blot results showed that the expression level of nuclear Nrf2 protein was increased in the kidney tissues of DN mice, demonstrating that the nuclear translocation of Nrf2 in the kidney tissues of DN mice significantly increased after high glucose induction and the stress injury caused by excessive ROS accumulation occurred. The expression levels of HO-1 and GPX4 increased with the increase in FST administration concentration, which confirmed that FST can play a role in activating Nrf2/HO-1 downstream antioxidant genes, scavenging excessive ROS, and enhancing the body's ability to resist oxidative stress.

Oxidative stress is an internal reaction caused by an imbalance between excessive production of reactive oxygen species and endogenous oxidative enzymes in organisms [29, 30]. Studies have indicated that ROS is also an important mechanism in the development of DN [31]. The hyperglycemic environment can lead to elevated ROS levels, resulting in DNA damage, protein and lipid oxidation, protein denaturation, etc. As an antioxidant, GSH prevents damage to important cellular components caused by reactive oxygen species, such as free radicals, peroxides, and lipid peroxides. MDA and SOD are often used as indicators of the level of ROS in the body. The decrease in antioxidant defenses can lead to an increase in oxidative stress, and an increase in oxidative stress leads to a depletion of antioxidant defenses like SOD and GSH, and the production of MDA will increase. When the production rate of MDA exceeds the clearance rate of SOD, it will damage the kidney and affect renal function [32]. Our results confirmed that the amount of serum SOD and GSH was increased and MDA was decreased following the treatment of FST in a dose-dependent manner, which indicated that FST could reduce ROS levels and alleviate oxidative stress-induced kidney damage.

Proteomics nowadays are widely used in pharmacological studies to discover new targets and signaling pathways. Several studies have shown that when cells are stimulated by peroxidation, Nrf2 translocates to the nucleus and also promotes the expression of multiple downstream target genes, such as NQO-1 and CAT, in addition to HO-1. The expression of these target genes is clearly shown in the differential protein heat map (Figure 4). NQO-1 and CAT protein expressions increased significantly in the DN model group mice, suggesting that the organism experienced oxidative stress. The expression of NQO-1 and CAT proteins in DN model mice increased significantly, suggesting oxidative stress, while the expression of target genes was significantly downregulated after FST administration, indicating that reactive oxygen species were reduced and renal injury was mitigated. The expression trend of desmin protein is also obvious in the figure, which corroborated the WB and fluorescence results of this experiment.

During drug metabolism, phase II detoxification enzymes such as NAD(P)H, GST, and GCL are regulated by Nrf2 [33]. The heatmap of differentially expressed proteins confirmed that FST activates antioxidant enzymes GST and Gclc in renal tissues, suggesting that FST activates the Nrf2 pathway, promotes the active expression of phase II detoxification enzymes, produces different degrees of oxidative stress inhibition, and protects renal cells from damage. Therefore, we speculate that FST may act on the Nrf2 signaling pathway to regulate these oxidative stress-related factors and jointly inhibit oxidative stress and promote phase II detoxification enzymes to reduce oxidative stress damage in the organism. In addition, it has been shown that FST can effectively prevent lipopolysaccharide-induced oxidative stress and inflammatory response, and the mechanism may be closely related to the inhibition of ROS/NF-*κ*B and activation of the Nrf2/HO-1 pathway [34]. FST also exerts neuroprotective effects on experimental diabetic neuropathy by regulating Nrf2 and NF-*κ*B pathways [35]. Moreover, FST has been shown to induce Nrf2-mediated HO-1 expression in human umbilical vein endothelial cells through PKC-*δ* and p38, thereby exerting a cytoprotective effect against oxidative stress [36].

In the next step, we will construct a siRNA that interferes with Nrf2 to verify the mechanism of action of FST on Nrf2. As for whether FST activates NRF2 by directly acting on Nrf2 or by regulating the upstream signaling pathway of NRF2, we need further verification.

Ferroptosis is a novel mode of cell death that is distinct from apoptosis, autophagy, and focal death. Ferroptosis is mainly characterized by massive deposition of iron ions, resulting in massive production of ROS, abnormal elevation of lipid oxidation radicals in the activated state of oxidative stress, and an imbalance of redox homeostasis, resulting in massive cell death [37]. Previous studies found that ACSL4 is involved in the synthesis of oxidizable membrane phospholipids, such as phosphatidylethanolamine and phosphatidylinositol, which promote lipid peroxidation of polyunsaturated fatty acids and thus participate in the onset of ferroptosis [38]. GPX4 acts as a sensor of oxidative stress and cell death signaling, and its reduced expression leads to a significant elevation of ROS in vivo, which is considered an important target for triggering the ferroptosis program. GPX4 is also a key factor in the ferroptosis chain. GSH is the main substrate of GPX4, and the decrease in GSH level will lead to the loss of GPX4 activity, and the inhibition of GPX4 will lead to the accumulation of lipid peroxides and consequently produce a large amount of ROS, leading to the occurrence of ferroptosis [39]. In this study, we detected that GPX4 expression was decreased and ACSL4 expression was significantly increased when podocytes were stimulated with different concentrations of high glucose, confirming the inhibition of intracellular lipid peroxide scavenging and intracellular reactive oxygen species accumulation. In contrast, the treatment of FST restored the abnormal increase in ACSL4 expression by upregulating GPX4 expression, scavenging a large accumulation of ROS and reducing the occurrence of ferroptosis in podocytes. Since no ferroptosis inhibitor/activator was used in this work, we will set up an ferroptosis inhibitor/activator for interference in the next experiments. We will also perform molecular docking experiments to investigate whether FST acts directly on NRF2 to regulate ferroptosis in podocytes, as well as to explore the possible binding sites at the protein and gene levels.

## 5. Conclusion

As the research has demonstrated, a type I DN model was successfully constructed using eNOS-/-mice after intraperitoneal injection of STZ. Our study shows that STZ affected the expression of antioxidant enzymes and cytokine mRNAs related to the Nrf2/HO-1/GPX4 signaling pathway in the kidney. However, FST could ameliorate DN-induced podocyte injury by modulating the Nrf2/HO-1/GPX4 signaling pathway.

In this study, we innovatively used a high-throughput approach to discuss the expression mechanisms of differential proteins and cross-validated them with the findings of others to further confirm the relationship between FST and the Nrf2/HO-1/GPX4 signaling pathway. These findings may provide new ideas and an experimental basis for the earlier stages of DN in the clinical setting and may also provide some inspiration for new therapeutic target research.

## Data Availability

The data supporting the findings of this data are available from the corresponding author upon reasonable request.

## Abbreviations

DN: Diabetic nephropathy
FST: Fisetin
HG: High glucose
STZ: Streptozotocin
Nrf2: Nuclear factor erythroid-2 related factor 2
HO-1: Heme oxygenase-1
GPX4: Glutathione peroxidase 4
ROS: Reactive oxygen species
GSH: Reduced glutathione
MDA: Malondialdehyde
SOD: Superoxide dismutase
ACSL4: Acyl-CoA synthetase long-chain family member 4
SCr: Serum creatinine
NC: Normal control
NQO-1: Quinone oxidoreductase 1
CAT: Catalase
GST: Glutathione S-transferase
GCL: Glutamate-cysteine ligase
siRNAs: Small interfering RNAs.
